# Combination of Taxanes, Cisplatin and Fluorouracil as Induction Chemotherapy for Locally Advanced Head and Neck Cancer: A Meta-Analysis

**DOI:** 10.1371/journal.pone.0051526

**Published:** 2012-12-07

**Authors:** Hao Qin, Jie Luo, Yuan-Ping Zhu, Hai-Li Xie, Wei-Qiang Yang, Wen-Bin Lei

**Affiliations:** 1 Department of Otorhinolaryngology, The First Affiliated Hospital of Sun Yat–sen University, Otorhinolaryngology Institute, Sun Yat–sen University, Guangzhou, China; 2 Department of Biostatistics and Epidemiology, School of Public Health, Sun Yat-sen University, Guangzhou, China; University Clinic of Navarra, Spain

## Abstract

**Background:**

Some investigations have suggested that induction chemotherapy with a combination of taxanes, cisplatin and fluorouracil (TPF) is effective in locally advanced head and neck cancer. However, other trials have indicated that TPF does not improve outcomes. The objective of this study was to compare the efficacy and safety of TPF with a cisplatin and fluorouracil (PF) regimen through a meta-analysis.

**Methods:**

Four randomized clinical trials were identified, which included 1,552 patients with locally advanced head and neck cancer who underwent induction chemotherapy with either a TPF or PF protocol. The outcomes included the 3-year survival rate, overall response rate and different types of adverse events. Risk ratios (RRs) and their 95% confidence intervals (CIs) were pooled using RevMan 5.1 software.

**Results:**

The 3-year survival rate (51.0% vs. 42.4%; *p* = 0.002), 3-year progression-free survival rate (35.9% vs. 27.2%; *p* = 0.007) and overall response to chemotherapy (72.9% vs. 62.1%; *p*<0.00001) of the patients in the TPF group was statistically superior to those in the PF group. In terms of toxicities, the incidence of febrile neutropenia (7.0% vs. 3.2%; *p* = 0.001) and alopecia (10.8% vs. 1.1%; *p*<0.00001) was higher in the TPF group.

**Conclusion:**

The TPF induction chemotherapy regimen leads to a significant survival advantage with acceptable toxicity rates for patients with locally advanced head and neck cancer compared with the PF regimen.

## Introduction

Head and neck cancer is one of commonest malignant tumors, with approximately 500,000 new cases diagnosed each year. Squamous-cell carcinoma is the predominant histological type. Unfortunately, the prognosis for this form of cancer is still dismal at present, and the recurrence rate ranges from 10 to 40%. This could be related to the fact that approximately 60% of cases initially present with advanced stage disease (stage III–IV). Only 30% to 50% of patients with locally advanced disease live for 3 years after standard therapy (surgery and irradiation), and 40% to 60% of them develop locoregional recurrences or distant metastases [1], [2], [3], [4], [5], [6].

Induction chemotherapy has been evaluated in clinical trials since the 1970s, and the cisplatin/fluorouracil (PF) regimen has been shown to be the most frequently used one, with slight but significant survival benefits. Therefore, induction chemotherapy has become a dominant community practice in the USA [7], [8], [9]. Recently, a series of clinical trials have suggested that taxanes (docetaxel or paclitaxel) plus the standard PF regimen (the TPF regimen) improves outcomes in locally advanced head and neck cancer [7], [10], [11], [12], whereas another trial has indicated that no improvement occurs [13]. Therefore, we decided to analyze these trials through a meta-analysis in order to obtain a higher statistical power. The main objective of this study was to evaluate the efficacy and safety of induction chemotherapy with the TPF regimen in locally advanced head and neck cancer compared to the PF regimen.

**Figure 1:**
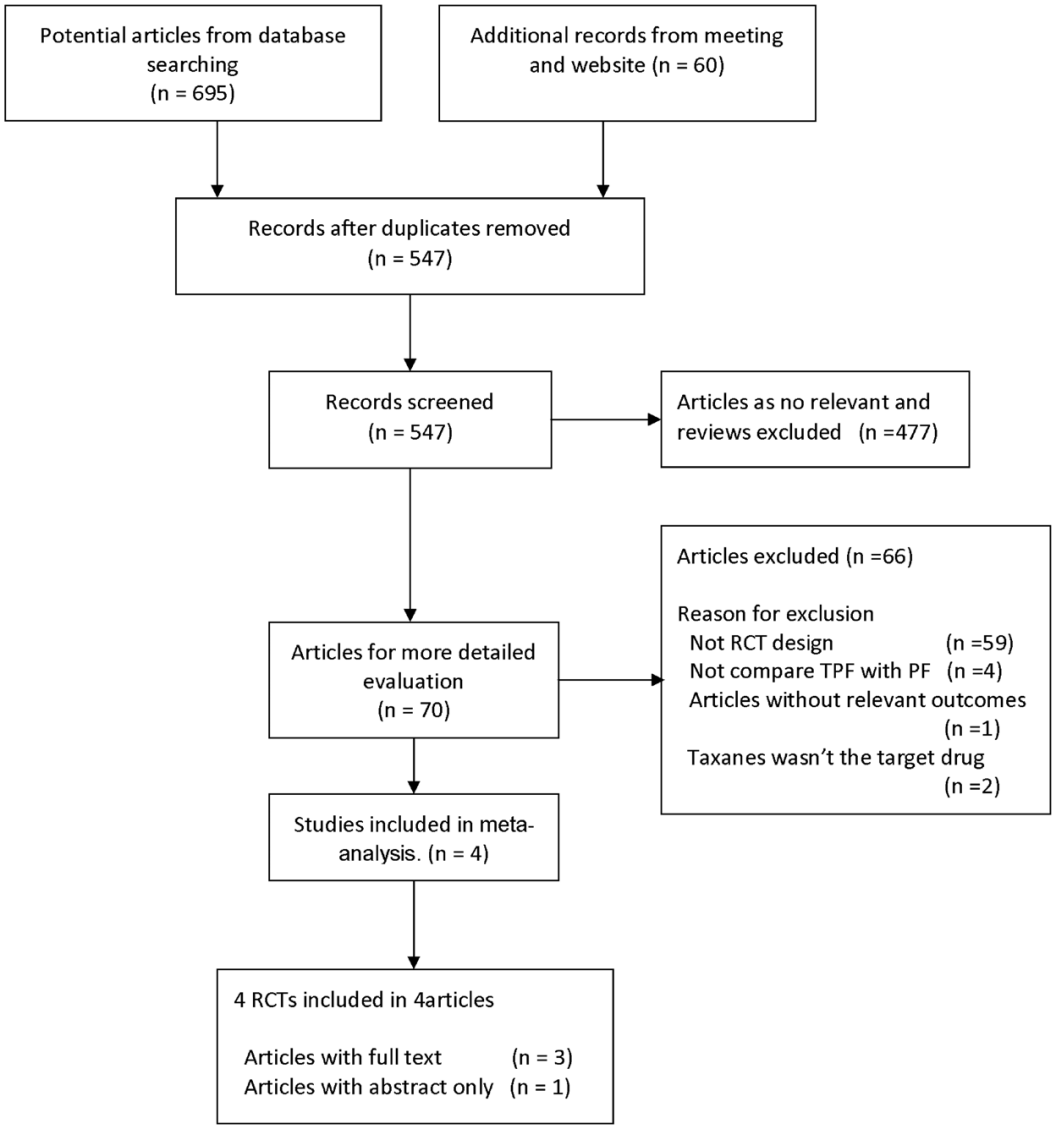
Flow diagram of the trials search and selection process.

## Materials and Methods

### Identification of Eligible Trials

PubMed, EMBASE, SpringerLink, MEDLINE, the Cochrane Center Register of Controlled Trials, the registry of the U.S. National Institutes of Health clinicaltrials.gov and the American Society of Clinical Oncology Annual Scientific Meeting (ASCO) databases were searched from their inception to May 2012, using the following search terms: “randomized”, “head and neck cancer”, “SCCHN”, “induction chemotherapy” and “taxanes or docetaxel or paclitaxel”. Clinical trials that met the following criteria were included: (1) a prospective randomized controlled trial (RCT); (2) patients that suffer squamous cell cancer of the head and neck (SCCHN) in stage III or IV without distant metastases; (3) the primary outcome was disease-free survival (DFS) or overall survival (OS); and (4) a comparison of the combined TPF induction chemotherapy regimen with the traditional PF regimen. To avoid publication bias, both published and unpublished trials were included. This review was conducted and reported according to the PRISMA (Preferred Reporting Items for Systematic Reviews and Meta-Analysis) Statement issued in 2009 [14].

**Table 1:** Characteristics of randomized controlled clinical trials in the meta-analysis.

Study	Treatments after induction chemotherapy	Sample size (TPF/PF)	Median age(year)	Performance status	Male (%)	Median follow up(month)	Quality score
Vermorken [16]	radiotherapy	177/181	TPF 53; PF 53	WHO-PS≤1	TPF 89.8; PF 89.5	32.5	3
Posner [17]	chemoradiotherapy	255/246	TPF 55; PF 56	WHO-PS≤1	TPF 84; PF 83	TPF 42; PF 41	3
Hitt [18]	chemoradiotherapy	189/193	TPF 56; PF 55	ECOG≤1	TPF 94; PF 94	23.2	3
Hitt [19]	chemoradiotherapy	155/156	TPF 58; PF 57	ECOG≤1	TPF 94; PF 93	67.6	2

WHO-PS, WHO performance status;Karnofsky PS, Karnofsky Performance Scale;ECOG, Eastern Cooperative Oncology Group performance status. Study quality was assessed on the 7-item Jadad scale, with a score range of 0 to 5; CI, continuous infusion.

### Data Extraction and Study Quality

Data extraction was conducted independently by three investigators. The following data was extracted from each eligible study: study design, year of publication, induction chemotherapy regimen, treatment after induction chemotherapy, sample size, median age, performance status, gender ratio, median follow-up, 3-year survival rate, 3-year progression-free survival rate, overall response to chemotherapy and any grade 3 or 4 toxicity (neutropenia, febrile neutropenia, stomatitis/mucositis, anemia, thrombocytopenia, infection, alopecia, lethargy, nausea and vomiting). The reporting quality of each clinical trial was assessed and calculated using the Jadad scale, including the presence of randomization, double-blinding, and withdrawals, by three independent evaluators [15]. A general quality score was assigned to each study as follows: 0 (non-randomized controlled trials), 1∼2 (low quality studies), and 3∼5 (high quality studies). Data were adjudicated by two additional investigators according to the original articles after data extraction and assessment.

**Figure 2:**
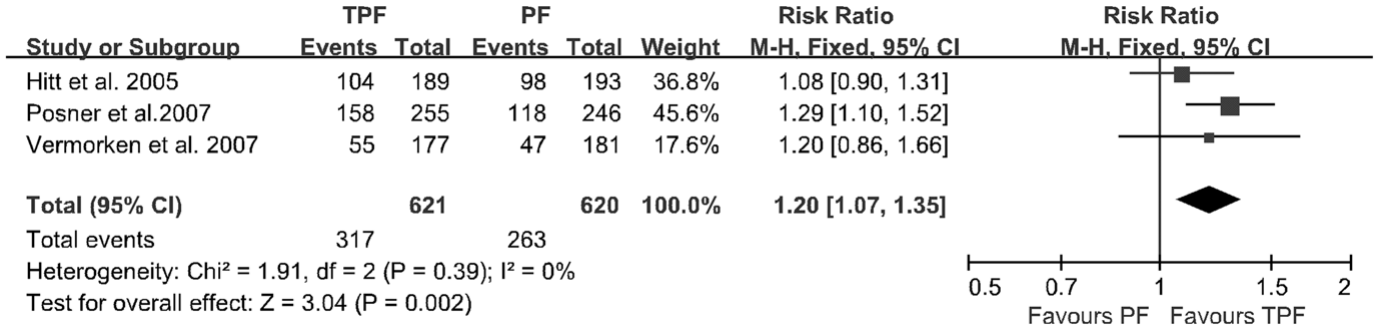
Forest plot of treatment effect on 3-year survival rate.

**Figure 3:**
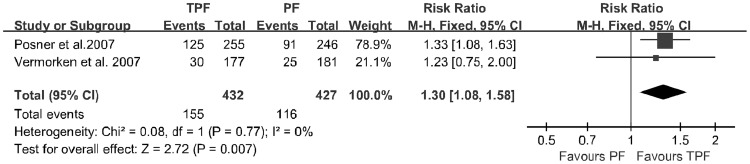
Forest plot of treatment effect on 3-year progression-free survival rate.

### Statistical Methods

Statistical analyses were performed using Review Manager 5.1 provided by The Cochrane Collaboration. The primary end point was the 3-year overall survival rate and overall response to chemotherapy and the secondary end points were grade 3–4 adverse events. For the calculation of risk ratios (RR), patients assigned to the TPF regimen were compared with those assigned to the PF regimen in the same trial.

**Figure 4:**
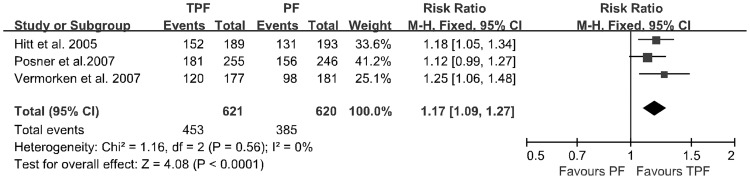
Forest plot of treatment effect on overall response to chemotherapy.

For the meta-analysis, both the fixed-effects model and a random-effects model were considered. For each meta-analysis, the Cochrane *Q* statistic and the *I^2^* value were calculated to assess the heterogeneity of the study. If a *p*-value was <0.1, the assumption of homogeneity was deemed invalid and the random-effects model was used after exploring the causes of heterogeneity; otherwise, the fixed-effects model was reported. The results of the meta-analysis were described by classical forest plots, the individual squares represent each study’s RR estimate. The lines extending from the squares represent the 95% confidence interval (CI) for the estimate. The size of the square represents the weight that the corresponding study exerts in the meta-analysis. Any potential publication bias was evaluated by inspecting funnel plots and the Begg-Mazumdar test [16].

**Figure 5:**
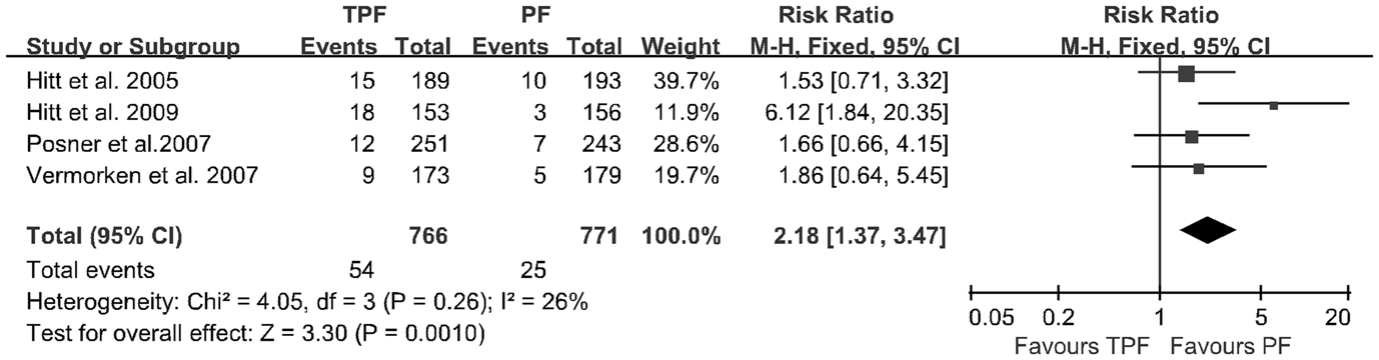
Forest plot of risk ratio on febrile neutropenia.

## Results

### Search Results

We identified 755 potentially relevant papers, of which 685 were excluded after their abstracts were screened.A flowchart summarizing search results is provided in Figure 1. Eventually, four clinical trials [17], [18], [19], [20] that included 1,552 patients were selected for the meta-analysis. These trials included both patients with resectable and unresectable disease. Their basic characteristics and detailed induction chemotherapy regimens were listed in Table 1.

**Figure 6:**
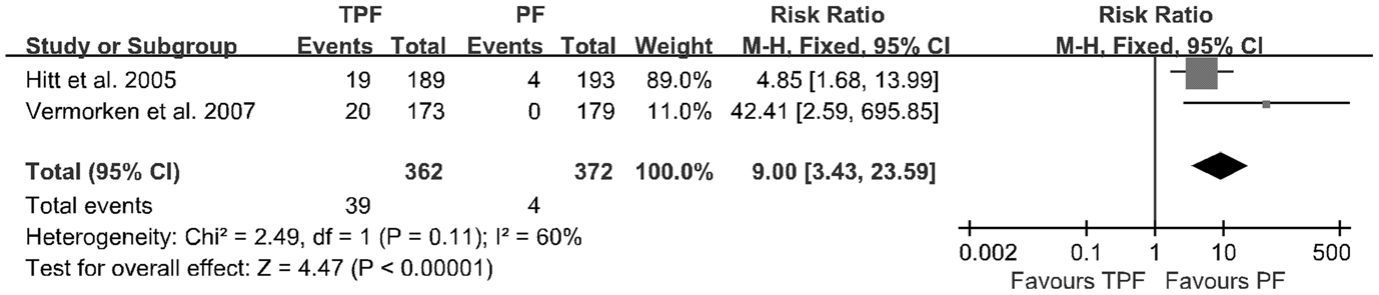
Forest plot of risk ratio of alopecia.

### Three-Year Survival Rate

Three of the four trials provided data regarding the 3-year survival rate. The TPF group included 621 patients with a 3-year survival rate of 51.0%, while the PF group included 620 patients a 3-year survival rate of 42.4%. The test for heterogeneity of the data yielded a *p*-value of 0.39; thus, we accepted the hypothesis of homogeneity. When combined, the data from the four trials yielded an estimated common RR of 1.20 (95% CI: 1.07∼1.35), and a significant effect on survival was found for the use of TPF for induction chemotherapy (*p* = 0.002; Figure 2).

**Table 2:** Summary of Grade 3–4 adverse events By Treatment.

Adverse effect	No. of PatientsWith Available Data	TPF arm (No./%)	PF arm (No./%)	Exact RR (95% CI)	*p*	*p* for Homogeneity
Neutropenia	1537	320/41.8	258/33.5	1.22(0.97–1.54)	0.09	0.03
Febrile neutropenia	1537	54/7	25/3.2	2.18(1.37–347)	0.001	0.26
Stomatitis/mucositis	1537	101/13.2	195/25.7	0.55(0.29–1.03)	0.06	<0.0001
Anemia	846	28/6.6	32/7	0.88(0.54–1.44)	0.62	0.27
Thrombocytopenia	1155	31/5.4	50/8.7	0.74(0.27–2.01)	0.55	0.01
Infection	846	18/4.2	16/3.8	1.14(0.59–2.20)	0.7	0.97
Alopecia	734	39/10.8	4/1.1	9.00(3.43–23.59)	<0.00001	0.11
Lethargy	846	10/2.4	13/2.4	0.76(0.34–1.72)	0.51	0.16
Nausea	846	15/3.5	26/6.2	0.34(0.03–4.11)	0.4	0.02
Vomiting	846	9/2.1	18/4.3	0.49(0.22–1.09)	0.08	0.11

### Three-year Progression-free Survival Rate

Two trials provided data regarding the 3-year progression-free survival rate. Patients treated with the TPF regimen had significantly longer 3-year progression-free survival rates (RR: 1.30; 95% CI: 1.08∼1.58; p = 0.007; incidence, 35.9% vs. 27.2%) and no significant heterogeneity was found among these studies (*p* = 0.77; Figure 3).

### Overall Response to Chemotherapy

Three trials provided the data for the overall response (WHO criteria) to chemotherapy. Among the 1,241 eligible patients (621 vs. 620 patients) in the three RCTs, patients treated with TPF had significantly more overall response rate (RR 1.17; 95% CI, 1.09∼1.27; *p*<0.00001; incidence, 72.9% vs. 62.1%) and no significant heterogeneity was found among these studies (*p* = 0.56; Figure 4).

### Toxicity

The data of adverse events were extracted from the four trials and analyzed by a meta-analysis. The results are described in Table 2. Patients treated with the TPF regimen had a significantly higher rate of grade 3 to 4 febrile neutropenia (7.0% vs. 3.2%; *p* = 0.001; Figure 5) and alopecia (10.8% vs. 1.1%; *p*<0.00001; Figure 6). Heterogeneity was found for some adverse events, which was possibly due to the use of different agents at various dosages in the four studies.

## Discussion

The outcomes of induction chemotherapy with cisplatin and fluorouracil for locally advanced head and neck cancer have been evaluated in clinical trials for more than two decades. A meta-analysis of trials using traditional PF regimen demonstrated a significant survival benefit for this approach over locoregional treatment alone in locally advanced disease [9]. Taxanes are a group of anticancer drugs that function by inducing tubulin polymerization to form extremely stable and nonfunctional microtubules. These agents can block the G2/M phases of the cell cycle, leading to mitotic arrest. In vitro studies suggest that they possibly play a role in radiation sensitization [21], [22]. During the 1990s, taxanes have shown great promise for the treatment of squamous cell carcinoma of the head and neck [23]. Therefore, investigators have started to add these agents in induction chemotherapy regimens. Several phase II and III trials have indicated that taxanes plus cisplatin and fluorouracil (TPF) might be more efficacious and cost-effective than the PF regimen [17], [18], [19], [20], [24], [25]. However, in 2009, Pointreau et al. [13] reported another phase III study that demonstrated no significant difference in the 3-year overall survival between regimens (60% in each arm), and the disease-free interval in patients suffering laryngeal or hypopharyngeal cancer. As this new TPF regimen remains controversial, we performed this meta-analysis to compare its efficacy and safety as the induction chemotherapy in patients with locally advanced head and neck cancer with the PF regimen.

Our meta-analysis indicated that the TPF induction chemotherapy regimen led to a significant improvement in the 3-year survival rate (8.6%), 3-year progression-free survival rate (8.7%) and overall response (10.8%) compared with the PF regimen. The TPF regimen improved the survival of patients with stage III and IV squamous cell cancer of the head and neck (SCCHN) without distant metastases. In terms of the long-term results of this new regimen, one of the four randomized trials demonstrated that the 5-year survival was 10% higher in patients treated with TPF, while the progression-free survival was also significantly improved (38.1 months vs. 13.2 months) [26]. The long-term results from the other three trials are not available at present. Therefore, we consider that the induction chemotherapy regimen of TPF may be superior to the PF regimen in terms of efficacy, and the long-term results of the four trials will be of interest in the future.

With regards to toxicities, it has been reported that the most common adverse event after taxane therapy is high grade neutropenia. Other toxicities include infusion-related hypersensitivity reactions, alopecia, neurotoxicity, mucositis, diarrhea and myalgias [27]. Our pooled analysis showed that the TPF regimen produced a higher rate of grade 3 to 4 febrile neutropenia (7.0% vs. 3.2%; *p* = 0.001) and alopecia (10.8% vs. 1.1%; *p*<0.00001) than PF, but did not lead to more infectious complications (4.2% vs. 3.8%; *p* = 0.70) if patients received prophylactic antibiotics. However, the incidences of neutropenia, stomatitis/mucositis, anemia, thrombocytopenia, lethargy, nausea and vomiting were not significantly different. It should be noticed that all of the four included studies were performed in non-Asian countries. Yano et al. reported that there is a significant difference in the incidence of docetaxel-induced severe neutropenia between Asian and non-Asian clinical studies. The studies performed in Asia showed an almost 19 times higher risk than the non-Asian studies [28]. Thus, the TPF regimen may lead to higher incidences of treatment failure and recurrence rates in Asian patients, Asian physicians and pharmacists should be aware of ethnic diversity in docetaxel toxicity. Infections can be prevented with the use of prophylactic antibiotics if awareness is improved, and the recovery from neutropenia can be accelerated after chemotherapy with the TPF regimen.

There were limitations in our study. Firstly, only four RCTs were included and not all individual patient data were available. Secondly, the characteristics of the included trials were varied in terms of the follow-up and dosages. Thirdly, although publication bias was not found according to the funnel plots and the Begg-Mazumdar test, the possibility of the omission of further ongoing or unpublished studies may exist at the time of the writing of this manuscript. These defects may affect the credibility of the results to some extent, and further analysis of increased numbers of multicenter RCTs with larger sample sizes will be necessary to confirm our findings.

Despite the limitations of our research, this meta-analysis demonstrates that the TPF induction chemotherapy regimen leads to a significant survival advantage with acceptable toxicities compared with the traditional PF regimen. In summary, the TPF induction chemotherapy regimen, followed by radiotherapy or concurrent chemoradiotherapy, should be considered as an efficient intervention in locally advanced head and neck cancer.

## Supporting Information

Figure S1 **PRISMA 2009 Flow Diagram.**
(DOC)

Checklist S1 **PRISMA 2009 Checklist.**
(DOC)
