# Supplementary material for: Combination of Taxanes, Cisplatin and Fluorouracil as Induction Chemotherapy for Locally Advanced Head and Neck Cancer: A Meta-Analysis
Source: PLoS One. 2012 Dec 7;7(12):e51526. doi: 10.1371/journal.pone.0051526 (PMC3517538; doi:10.1371/journal.pone.0051526)
Supplement: Figure S1 — PRISMA 2009 Flow Diagram. (DOC) [file pone.0051526.s001.doc]

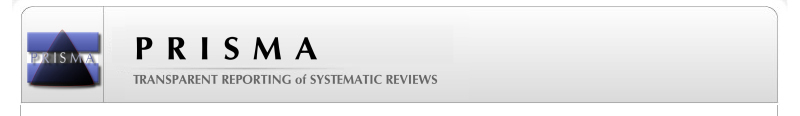
**PRISMA 2009 Flow Diagram**

**Screening**

**Included**

**Eligibility**

**Identification**

Potential articles from database searching
(n = 695)

Additional records from meeting and website (n = 60)

Records after duplicates removed
(n = 547)

Records screened
(n = 547)

Articles as no relevant and reviews excluded (n =477)

Articles for more detailed evaluation
(n = 70)

Articles excluded (n =66)

Reason for exclusion

Not RCT design (n =59)

Not compare TPF with PF  (n =4) Articles without relevant outcomes

(n =1)

Taxanes wasn’t the target drug

(n =2)

Studies included in meta-analysis. (n = 4)

4 RCTs included in 4articles

Articles with full text (n = 3)

Articles with abstract only (n = 1)
