# Supplementary material for: Combination of Taxanes, Cisplatin and Fluorouracil as Induction Chemotherapy for Locally Advanced Head and Neck Cancer: A Meta-Analysis
Source: PLoS One. 2012 Dec 7;7(12):e51526. doi: 10.1371/journal.pone.0051526 (PMC3517538; doi:10.1371/journal.pone.0051526)
Supplement: Checklist S1 — PRISMA 2009 Checklist. (DOC) [file pone.0051526.s002.doc]

| **Section/topic** | **#** | **Checklist item** | **Reported on page #** |
| --- | --- | --- | --- |
| **TITLE** | | |  |
| Title | 1 | Combination of Taxanes, Cisplatin and Fluorouracil as Induction Chemotherapy for Locally Advanced Head and Neck Cancer: a Meta-Analysis. | 1 |
| **ABSTRACT** | | |  |
| Structured summary | 2 | Background. Some investigations have proven that induction chemotherapy through combination of taxanes, cisplatin and fluorouracil (TPF) is effective in locally advanced head and neck cancer. The objective of this study is to compare its efficacy and safety with the regimen of cisplatin and fluorouracil (PF) through meta-analysis.  Methods. Four randomized clinical trials were identified, including 1552 patients with locally advanced head and neck cancer undergone induction chemotherapy through combination of taxanes, cisplatin and fluorouracil. The outcomes included 3-year survival rate, overall response rate, and different types of adverse events. Risk ratios (RRs) and their 95% confidence intervals (CIs) were pooled using RevMan 5.1 software.  Results. The 3-year survival rate(51.0% vs. 42.4%；p= 0.002 ), 3-year progression-free survival rate (35.9 % vs. 27.2 %; p=0.007) and overall response to chemotherapy（72.9% vs. 62.1%；p<0.00001）of the patients in TPF group was statistically superior to those in cisplatin and fluorouracil(PF) group .In terms of toxicities, the incidence of febrile neutropenia (7.0% vs. 3.2%; p=0.001) and alopecia (14.1% vs. 1.3%; p<0.00001) was higher in TPF group.  Conclusion. The induction chemotherapy regimen of TPF leads to a significant survival advantage with acceptable toxicities for locally advanced head and neck cancer patients comparing with PF regimen. | 1 |
| **INTRODUCTION** | | |  |
| Rationale | 3 | Head and neck cancer is one of common malignant tumors, Unfortunately, its prognosis is still dismal at present.Induction chemotherapy has been evaluated in clinical trials since 1970s and the regimen of cisplatin/fluorouracil (PF) was the most frequently used induction chemotherapy regimen. Recently some clinical trials suggest that taxanes (docetaxel or paclitaxel) plus standard PF can improve the survival, whereas some trials indicate there is no improvement.However, all of these trials were lack of statistical power due to small sample size. This new TPF regiment remains controversial | 1 |
| Objectives | 4 | The main objective of this study is to evaluate the efficacy and safety of induction chemotherapy with TPF regimen in locally advanced head and neck cancer comparing with PF. | 1 |
| **METHODS** | | |  |
| Protocol and registration | 5 | This research use the PRISMA (Preferred Reporting Items for Systematic Reviews and Meta-Analyses) statement as a guide.(available at： http://www.prisma-statement.org) | 2 |
| Eligibility criteria | 6 | Prospective randomized controlled trials (RCTs),published or unpublished. | 2 |
| Information sources | 7 | Clinical trials were searched on PubMed, EMBASE, SpringerLink, MEDLINE, Cochrane Library and the registry of the U.S. National Institutes of Health clinicaltrials.gov from inception to May 2012. | 2 |
| Search | 8 | Clinical trials were searched using the search terms of "randomized," "Head and Neck Cancer," "SCCHN," "induction chemotherapy," and "taxanes or docetaxel or paclitaxel". | 2 |
| Study selection | 9 | Clinical trials that met the following criteria were included: (1) prospective randomized controlled trial (RCT); (2) patients suffering squamous cell cancer of the head and neck (SCCHN) in stage Ⅲ or Ⅳ without distant metastases; and (3) comparison of the combined induction chemotherapy regimen of cisplatin, fluorouracil, and taxanes (TPF) with the traditional one of cisplatin plus fluorouracil. To avoid publication bias, both published and unpublished trials were included. | 2 |
| Data collection process | 10 | Data extraction were conducted independently by 3 investigators and the report quality of clinical trials was assessed and calculated using the Jadad scale, including randomization, double-blinding, and withdrawals by 3 independent evaluators. Data were adjudicated by 2 additional investigators according to the original articles after data extraction and assessment. | 2 |
| Data items | 11 | Induction chemotherapy regimens, treatment after induction chemotherapy, sample size, median age of patients, performance status of patients, gender ratio of patients, median follow-up, trial quality, 3-year survival rate, 3-year progression-free survival rate, overall response to chemotherapy, toxicity(neutropenia, febrile neutropenia, stomatitis/mucositis, anemia, thrombocytopenia, infection, alopecia, lethargy, nausea, vomiting). | 2 |
| Risk of bias in individual studies | 12 | The report quality of clinical trials was assessed and calculated using the Jadad scale, including randomization, double-blinding, and withdrawals by 3 independent evaluators. A general quality score was assigned to each study: 0 (non-randomized controlled trials), 1~2 (low quality studies), 3~5 (high quality studies). | 2 |
| Summary measures | 13 | risk ratio | 2 |
| Synthesis of results | 14 | For the calculation of risk ratio (RR), patients assigned to TPF regimen were compared with those assigned to PF regimen in the same trial. For meta-analysis, both the fixed-effects model and a random-effects model were considered. For each meta-analysis, the Cochran Q statistic and I2 statistics were calculated to assess the heterogeneity. If p value was <0.1, the assumption of homogeneity was deemed invalid and the random-effects model was used after exploring the causes of heterogeneity; otherwise, the fixed-effects model was reported. The results of meta-analysis were described by classical Forest plots, with point estimates and 95% conference interval (CI) for each trial and overall; size of the squares is proportional to study size. The potential publication bias was evaluated by inspecting funnel plots and the Begg-Mazumdar test. | 2 |

| **Section/topic** | **#** | **Checklist item** | **Reported on page #** |
| --- | --- | --- | --- |
| Risk of bias across studies | 15 | The potential publication bias was evaluated by inspecting funnel plots and the Begg-Mazumdar test.. | 2 |
| Additional analyses | 16 | There is no additional analyse in this meta-analysis. |  |
| **RESULTS** | | |  |
| Study selection | 17 | 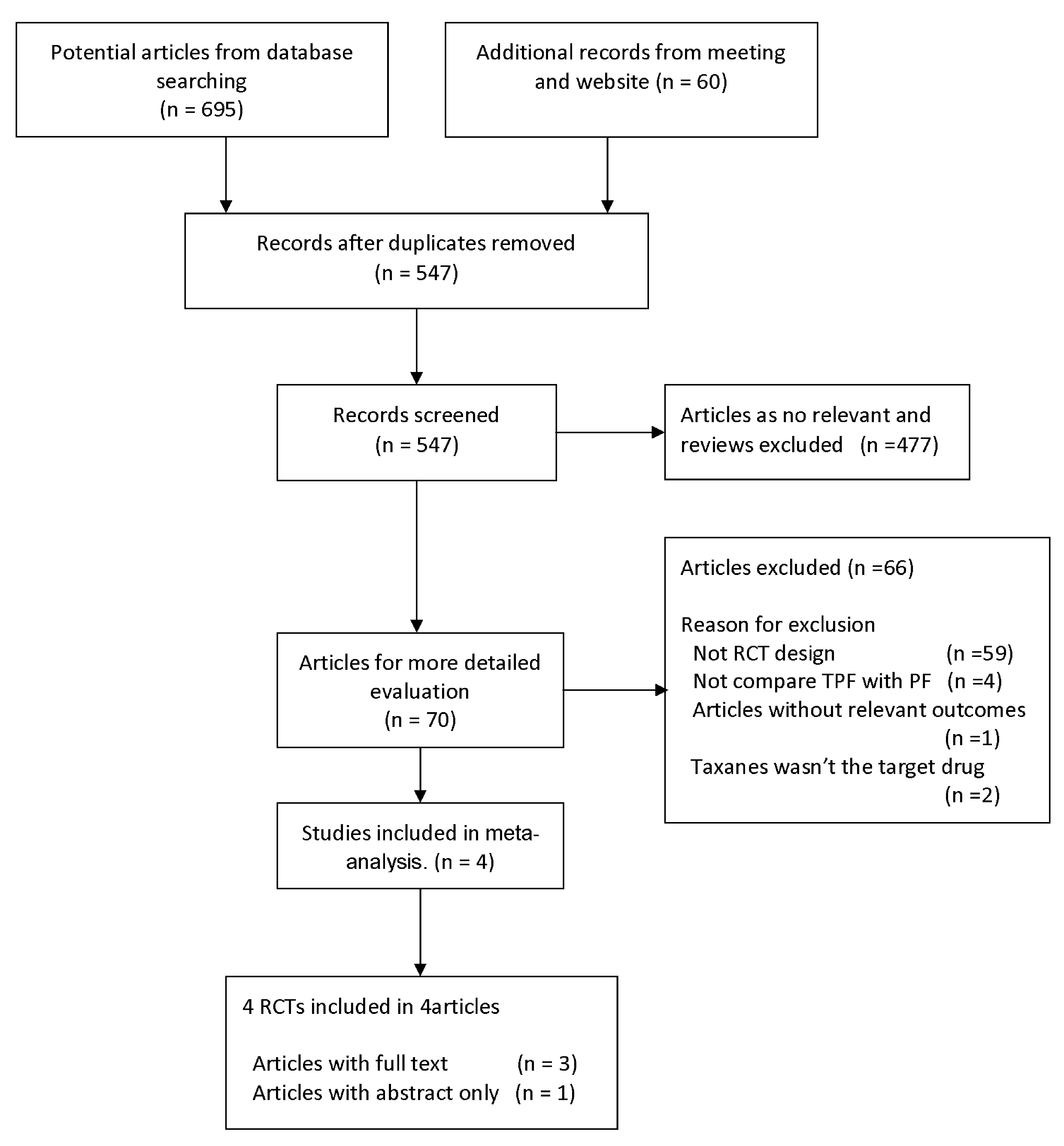 | 2 |
| Study characteristics | 18 | 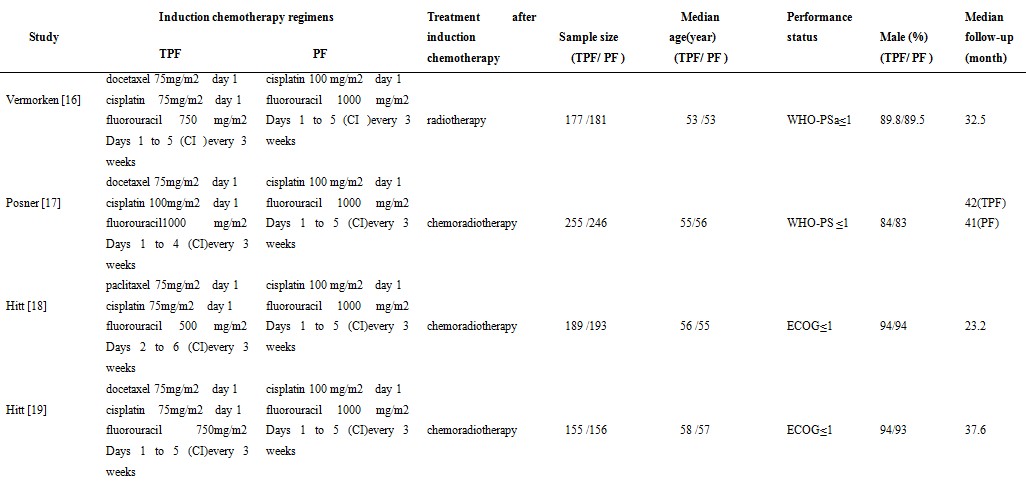  16. Posner MR, Hershock DM, Blajman CR, Mickiewicz E, Winquist E, et al. (2007) Cisplatin and fluorouracil alone or with docetaxel in head and neck cancer. N Engl J Med 357: 1705-1715.  17. Vermorken JB, Remenar E, van Herpen C, Gorlia T, Mesia R, et al. (2007) Cisplatin, fluorouracil, and docetaxel in unresectable head and neck cancer. N Engl J Med 357: 1695-1704.  18. Hitt R, Lopez-Pousa A, Martinez-Trufero J, Escrig V, Carles J, et al. (2005) Phase III study comparing cisplatin plus fluorouracil to paclitaxel, cisplatin, and fluorouracil induction chemotherapy followed by chemoradiotherapy in locally advanced head and neck cancer. J Clin Oncol 23: 8636-8645.  19. Hitt R, Grau JJ, Lopez-Pousa A, Berrocal A, Giron CG, et al. (2009) Final results of a randomized phase III trial comparing induction chemotherapy with cisplatin/5-FU or docetaxel/cisplatin/5-FU follow by chemoradiotherapy (CRT) versus CRT alone as first-line treatment of unresectable locally advanced head and neck cancer (LAHNC). | 6 |
| Risk of bias within studies | 19 | | Study | Quality score （Jadad scale） | Risk of bias | | --- | --- | --- | | Vermorken [16] | 3 | Not a double-blinding study ,the dosages of cisplatin and fluorouracil are different | | Posner [17] | 3 | Not a double-blinding study | | Hitt [18] | 3 | Not a double-blinding study ,the dosages of cisplatin and fluorouracil are different | | Hitt [19] | 2 | Not a double-blinding study ,the dosages of cisplatin and fluorouracil are different |   Citations see Item 18 | 6 |
| Results of individual studies | 20 | **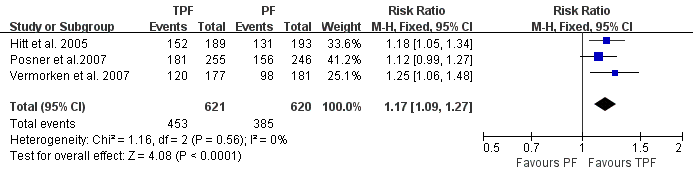**  **Fig 2. Forest plot of treatment effect on 3-year survival rate.**  **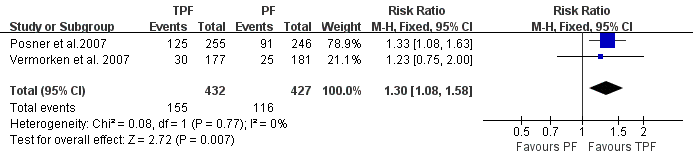**  **Fig 3. Forest plot of treatment effect on 3-year progression-free survival rate.**  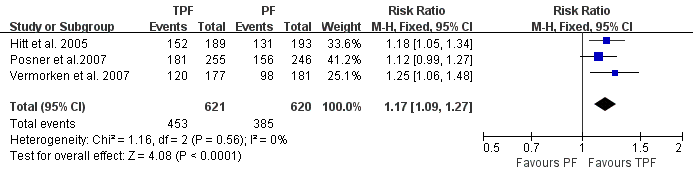  **Fig 4. Forest plot of treatment effect on overall response to chemotherapy.**  **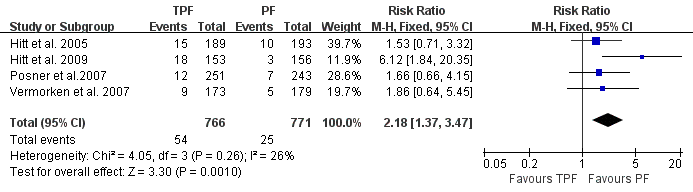**  **Fig 5. Forest plot of risk ratio on febrile** **neutropenia.**  **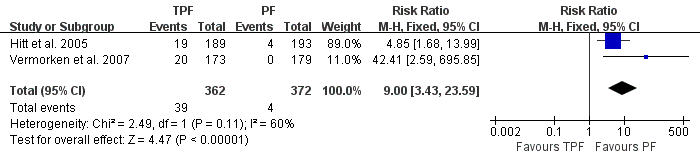**  **Fig 6. Forest plot of risk ratio of alopecia.** | 3 |
| Synthesis of results | 21 | **3-year survival rate.** RR=1.20, with a 95% CI of 1.07~1.35, and a significant effect of the TPF group for induction chemotherapy was found (*p*= 0.002; Fig. 2). No significant heterogeneity was found among these studies(test for heterogeneity of the data yielded *p*=0.39)  **3-year progression-free survival rate.** RR=1.30; 95%CI, 1.08~1.58; p=0.007; incidence, 35.9 % vs. 27.2 %) and no significant heterogeneity was found among these studies (p=0.77; Fig.3).  **Overall response to chemotherapy.** RR=1.17; 95%CI, 1.09~1.27; *p*<0.00001; incidence, 72.9 % vs. 62.1 %，no significant heterogeneity was found among these studies (*p*=0.56; Fig.4).  **Toxicity.** Patients treated with TPF regimen had significantly more grade 3 to 4 febrile neutropenia (7.0% vs. 3.2%; *p*=0.001; Fig.5), alopecia (10.8% vs. 1.1%; *p*<0.00001; Fig.6). Heterogeneity was found for some adverse events, possibly due to the difference between doses. | 3 |
| Risk of bias across studies | 22 | Publication bias was not found according to funnel plots and the Begg-Mazumdar test, | 4 |
| Additional analysis | 23 | There is no additional analyse in this meta-analysis. |  |
| **DISCUSSION** | | |  |
| Summary of evidence | 24 | Our meta-analysis indicates that the TPF induction chemotherapy regimen leads to significantly improvement in 3-year survival rate (8.6%), 3-year progression-free survival rate (8.7%) and overall response (10.8%) when compared with PF. Our pooled analysis showed that the TPF regimen produced more grade 3 to 4 febrile neutropenia (7.0% vs. 3.2%; *p*=0.001) and alopecia (10.8% vs. 1.1%; *p*<0.00001) than PF but did not lead to more infectious complications (4.2% vs. 3.8%; *p*=0.70) if patients received prophylactic antibiotics. | 4 |
| Limitations | 25 | There were limitations in our study. First, there were only 4 RCTs included and not all individual patient data were available. Second, the characteristics of the included trials were varied in the follow-up and dosage. Third, although publication bias was not found according to funnel plots and the Begg-Mazumdar test, possibility of ongoing or unpublished studies may still exist at the time of the writing of this manuscript. These defects may affect the credibility of the results to some extent, and further analysis of more multicenter RCTs with larger sample size is needed to confirm our findings. | 4 |
| Conclusions | 26 | TPF induction chemotherapy regimen leads to a significant survival advantage with acceptable toxicities as compared with PF regimen. | 4 |
| **FUNDING** | | |  |
| Funding | 27 | Funding: National Nature Science Foundation of China (81072224), a grant from the Sun yat-sen university young teachers cultivation project（10ykpyl0 ）and a grant sponsored by Guangdong Provincial Science and technology projects (2009B030801109). The funders had no role in the study design, data collection, analysis, decision to publish or preparation of the manuscript. The corresponding author had full access to all the data in the study and had the final responsibility for the decision to submit for publication | 4 |

*From:*  Moher D, Liberati A, Tetzlaff J, Altman DG, The PRISMA Group (2009). Preferred Reporting Items for Systematic Reviews and Meta-Analyses: The PRISMA Statement. PLoS Med 6(6): e1000097. doi:10.1371/journal.pmed1000097

For more information, visit: **www.prisma-statement.org**.
